# Supplementary material for: Biomarkers, Master Regulators and Genomic Fabric Remodeling in a Case of Papillary Thyroid Carcinoma
Source: Genes (Basel). 2020 Sep 2;11(9):1030. doi: 10.3390/genes11091030 (PMC7565446; doi:10.3390/genes11091030)
Supplement: Supplementary file 1 [file genes-11-01030-s001.pdf]

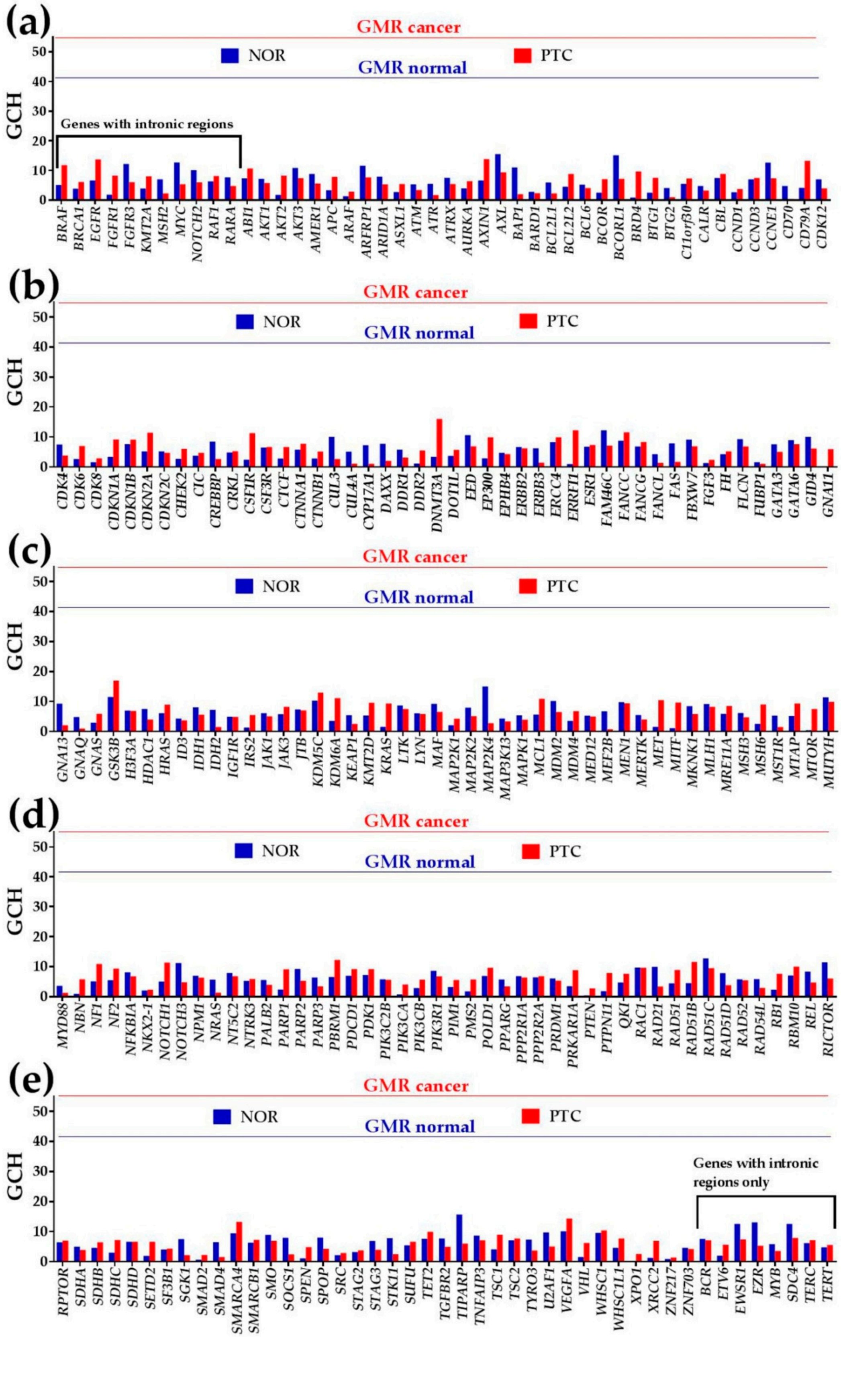
(a)
GMR cancer
NOR
PTC
GMR normal
GCH
0 10 20 30 40 50
Genes with intronic regions
BRAF BRCA1 EGFR FGFR1 FGFR3 KMT2A MSH2 MYC NOTCH2 RAF1 RARA ABI1 AKT1 AKT2 AKT3 AMER1 APC ARAF ARFRP1 ARID1A ASXL1 ATM ATR ATRX AURKA AXIN1 AXL BAP1 BARD1 BCL2L1 BCL2L2 BCL6 BCOR BCORL1 BRD4 BTG1 BTG2 C11orf30 CALR CBL CCND1 CCND3 CCNE1 CD70 CD79A CDK12
(b)
GMR cancer
NOR
PTC
GMR normal
GCH
0 10 20 30 40 50
CDK4 CDK6 CDK8 CDKN1A CDKN1B CDKN2A CDKN2C CHEK2 CIC CREBBP CRKL CSF1R CSF3R CTCF CTNNA1 CTNNB1 CUL3 CUL4A CYP17A1 DAXX DDR1 DDR2 DNMT3A DOT1L EED EP300 EPHB4 ERBB2 ERBB3 ERCC4 ERRFI1 ESR1 FAM46C FANCC FANCG FANCL FAS FBXW7 FGF3 FH FLCN FUBP1 GATA3 GATA6 GID4 GNA11
(c)
GMR cancer
NOR
PTC
GMR normal
GCH
0 10 20 30 40 50
GNA13 GNAQ GNAS GSK3B H3F3A HDAC1 HRAS ID3 IDH1 IDH2 IGF1R IRS2 JAK1 JAK3 JTB KDM5C KDM6A KEAP1 KMT2D KRAS LTK LYN MAF MAP2K1 MAP2K2 MAP2K4 MAP3K13 MAPK1 MCL1 MDM2 MDM4 MED12 MEF2B MEN1 MERTK MET MITF MKNK1 MLH1 MRE11A MSH3 MSH6 MST1R MTAP MTOR MUTYH
(d)
GMR cancer
NOR
PTC
GMR normal
GCH
0 10 20 30 40 50
MYD88 NBN NF1 NF2 NFKBIA NKX2-1 NOTCH1 NOTCH3 NPM1 NRAS NT5C2 NTRK3 PALB2 PARP1 PARP2 PARP3 PBRM1 PDCD1 PDK1 PIK3C2B PIK3CA PIK3CB PIK3R1 PIM1 PMS2 POLD1 PPARG PPP2R1A PPP2R2A PRDM1 PRKAR1A PTEN PTPN11 QKI RAC1 RAD21 RAD51 RAD51B RAD51C RAD51D RAD52 RAD54L RB1 RBM10 REL RICTOR
(e)
GMR cancer
NOR
PTC
GMR normal
GCH
0 10 20 30 40 50
Genes with intronic regions only
RPTOR SDHA SDHB SDHC SDHD SETD2 SF3B1 SGK1 SMAD2 SMAD4 SMARCA4 SMARCB1 SMO SOCS1 SPEN SPOP SRC STAG2 STAG3 STK11 SUFU TET2 TGFBR2 TIPARP TNFAIP3 TSC1 TSC2 TYRO3 U2AF1 VEGFA VHL WHSC1 WHSC1L1 XPO1 XRCC2 ZNF217 ZNF703 BCR ETV6 EWSR1 EZR MYB SDC4 TERC TERT

Figure S1. GCH scores of the genes included in FoundationOne®CDx assay. The assay explores genes with full coding exonic regions for the detection of substitutions, insertion-deletions (indels), and copy-number alterations (CNAs), genes with select intronic regions for the detection of gene rearrangements, one gene with a promoter region and one non-coding RNA gene. Several genes (*BRAF, BRCA1, EGFR, FGFR1/3, KMT2A, MSH2, MYC. NOTCH2, RAF1, RARA*) have both exonic and intronic regions used for detection.
